# Speaking valves in tracheostomised ICU patients weaning off mechanical ventilation - do they facilitate lung recruitment?

**DOI:** 10.1186/s13054-016-1249-x

**Published:** 2016-04-01

**Authors:** Anna-Liisa Sutt, Lawrence R. Caruana, Kimble R. Dunster, Petrea L. Cornwell, Chris M. Anstey, John F. Fraser

**Affiliations:** Critical Care Research Group, The Prince Charles Hospital, Brisbane, Australia; School of Medicine, University of Queensland, Brisbane, Australia; Speech Pathology Department, The Prince Charles Hospital, Brisbane, Australia; Physiotherapy Department, The Prince Charles Hospital, Brisbane, Australia; Science & Engineering Faculty, Queensland University of Technology, Brisbane, Australia; Allied Health Collaborative, Metro North HHS, Brisbane, Australia; School of Applied Psychology, Menzies Health Institute Queensland, Griffith University, Brisbane, Australia; Critical Care Research Group, Sunshine Coast University Hospital, Brisbane, Australia

**Keywords:** Mechanical ventilation, Tracheostomy, Communication, FRC, Speaking valve, Lung recruitment

## Abstract

**Background:**

Patients who require positive pressure ventilation through a tracheostomy are unable to phonate due to the inflated tracheostomy cuff. Whilst a speaking valve (SV) can be used on a tracheostomy tube, its use in ventilated ICU patients has been inhibited by concerns regarding potential deleterious effects to recovering lungs. The objective of this study was to assess end expiratory lung impedance (EELI) and standard bedside respiratory parameters before, during and after SV use in tracheostomised patients weaning from mechanical ventilation.

**Methods:**

A prospective observational study was conducted in a cardio-thoracic adult ICU. 20 consecutive tracheostomised patients weaning from mechanical ventilation and using a SV were recruited. Electrical Impedance Tomography (EIT) was used to monitor patients’ EELI. Changes in lung impedance and standard bedside respiratory data were analysed pre, during and post SV use.

**Results:**

Use of in-line SVs resulted in significant increase of EELI. This effect grew and was maintained for at least 15 minutes after removal of the SV (*p < 0.001*). EtCO_2_ showed a significant drop during SV use *(p = 0.01)* whilst SpO_2_ remained unchanged. Respiratory rate (RR (breaths per minute)) decreased whilst the SV was in situ (*p <0.001*), and heart rate (HR (beats per minute)) was unchanged. All results were similar regardless of the patients’ respiratory requirements at time of recruitment.

**Conclusions:**

In this cohort of critically ill ventilated patients, SVs did not cause derecruitment of the lungs when used in the ventilator weaning period. Deflating the tracheostomy cuff and restoring the airflow via the upper airway with a one-way valve may facilitate lung recruitment during and after SV use, as indicated by increased EELI.

**Trial registration:**

Anna-Liisa Sutt, Australian New Zealand Clinical Trials Registry (ANZCTR). ACTRN: ACTRN12615000589583. 4/6/2015.

## Background

Invasively ventilated patients are unable to phonate due to either the endotracheal tube positioning through the vocal folds, or when ventilating through the tracheostomy, the air bypassing the vocal folds. Speaking valves (SVs) can be used in-line with mechanical ventilation, but use of these requires deflation of the tracheostomy cuff [1]. Cuff deflation causes a leak in the ventilator circuit, which has been considered detrimental to patients’ ventilation, and potentially deleterious to weaning.

The key concern raised by physicians is that by deflating the cuff, and thus, losing positive end-expiratory pressure (PEEP) this could lead to loss of lung volume through alveolar collapse. It has been demonstrated that loss of PEEP in other events such as suctioning [2, 3] and ventilator disconnection [4] causes loss of lung volume. Current data indicate that “open lung ventilatory strategies” minimise ventilator-induced lung injury [5]. Hence, practices that may cause loss of lung volume must be used with some degree of caution.

One small case series has described the apparently safe use of SVs during weaning from mechanical ventilation [6]. Another study found no significant difference in ventilator weaning and decannulation times post the introduction of in-line SVs into an adult intensive care unit (ICU) [7, 8]. Whilst these studies provide preliminary clinical support for use of in-line SVs with tracheostomised mechanically ventilated patients, there are no physiological data to prove or allay fears.

Currently there are no data on the effect of SVs on end-expiratory lung volume (EELV), a critical point when the lungs are at most risk of collapsing. An SV is a one-way valve that allows for inspiration via the tracheostomy tube whilst expiration is redirected to the upper airway via the vocal folds, enabling phonation [1] and restored upper airway resistance. Hence, it can be considered functionally as a PEEP valve on the tracheostomy. As there is no airflow back into the ventilator tubing with the one-way valve, current in situ monitoring of ventilation with standard bedside equipment provides the clinician with limited information on ventilation. While computerised tomography or magnetic resonance imaging may be able to provide this information, the repeated use these imaging procedures could be seen as ethically unjustifiable, expensive, possibly requiring a level of sedation, and putting patients at risk with the transfer outside of the ICU environment [9, 10].

Electrical impedance tomography (EIT) is a radiation-free real-time bedside imaging tool capable of measuring the air movement in and out of the thorax [11–14]. It has been observed as being a safe, reliable and reproducible technique to assess regional ventilation in the lung, specifically during recruitment manoeuvres [3]. In the future it may be possible for absolute EIT to directly measure EELV but current time-differencing systems rely on measuring the difference between end-inspiratory lung impedance and EELI to measure tidal variation of impedance and changes in EELI [12]. There is linear correlation between changes in the EELI and changes in EELV [15–17], although this relationship tends to overestimate changes in EELV [16]. A limitation of time-differencing EIT is that it is unable to detect the pre-existing EELI [18, 19], which means it can only detect changes in EELI if the device remains in situ and running between readings [15, 18–20]. Researchers, however, have successfully used EIT to detect changes in EELI due to various clinical interventions such as suctioning, position change, and changes in PEEP [13, 16, 20–23].

The aim of this study was to assess the effect of SVs on EELI. Based on the findings of prior case studies it was hypothesised that there is an increase in global EELI with the SV in situ when patients are performing trials off the ventilator (i.e., on 50 L of 40 % oxygen via the tracheostomy). This may potentially be similar when patients are constantly supported by mechanical ventilation, given restored physiological PEEP. Secondary aims included determining the effects of SV on the patient’s respiratory rate (RR), heart rate (HR), oxygen saturation (SpO_2_) and end-tidal carbon dioxide (EtCO_2_). The potential effect on respiratory mechanics of talking versus quietly breathing with the SV in situ, and the effects of the SV and its dependence on the patients’ ventilatory requirements at the time, were also investigated.

## Methods

Following human ethics approval by the Institutional Review Board (HREC/13/QPCH/95) a prospective observational study (ACTRN12615000589583) using a repeated measures design was conducted. The study took place in a primarily cardiothoracic ICU at a metropolitan tertiary teaching hospital. Consecutive patients who were tracheostomised and being weaned from mechanical ventilation, from November 2013 to December 2014, were considered for inclusion in the study if they were tolerating a SV for a minimum of 30 minutes, as jointly assessed by a speech pathologist and a physician. Patients were excluded if they had significant language or cognitive deficits, or were not suitable to wear an EIT belt (i.e., patients with ventricular assist devices, open chest, extensive sternal dressings/drains or those dependant on cardiac pacing). In total 20 patients were recruited into two groups: 1) 10 patients on pressure support ventilation (PSV) and 2) 10 patients having trial periods off mechanical ventilation (and transferred onto high-flow or low-flow oxygen via the tracheostomy). All patients provided written informed consent, or for those unable to sign for written consent, the consent was provided by a legally authorised person (e.g., family member) or by the patient’s nurse witnessing verbal consent. The study was conducted in accordance with the ethical standards laid down in the 1964 Declaration of Helsinki.

### Measures

Following informed consent, patients were enrolled in the study. EIT (Pulmovista, Draeger Medical, Lubeck, Germany) measurements were taken continuously for 60 minutes with the frame rate set to 10 Hz to give the EELI per breath. Transitions to and from SV were followed by 15-minute periods, to allow for stabilisation [24].

Set ventilator-delivered PEEP and fraction of inspired oxygen (FiO_2_) data were collected from the ventilator (Puritan Bennett 840, Covidien, Dublin, Ireland). HR and SpO_2_ were measured with pulse oximeter (504, Criticare systems, Waukesha, WI, USA). Airway pressure (P_aw_) was measured directly via a neonatal feeding catheter (6 F) introduced through the Luer port of an adaptor (Ikaria, Hampton, NJ, USA) advanced to lie just distal to the tracheostomy cannula in the trachea, and measured with a pressure transducer (PPT, Honeywell, Morris Plains, NJ, USA). Oximeter and pressure data were collected at 200 Hz (PowerLab, AD Instruments, Sydney, NSW, Australia). EtCO_2_ was sampled from the feeding tube and measured (Marquette Solar 8000, GE Healthcare, Little Chalfont, UK). EtCO_2_ was measured continuously throughout the 60 minutes apart from 2 minutes before, during and after SV use when continuous P_aw_ measurements were taken through the same catheter. There was no flow through the catheter during pressure measurements, to ensure highest possible fidelity. All data were collected on a breath-to-breath basis using custom-written software.

### Procedure

The patients were positioned either in bed at 45 degrees or in a straight-backed chair with the EIT electrode belt around their chest at the level of the fifth to sixth anterior intercostal space. As patient position has been shown to have an impact on ventilation distribution [13], we ensured that there were no significant changes in patient positioning throughout the data collection. A neonatal feeding catheter was inserted as described above and the pulse oximeter was positioned on the finger.

Fifteen minutes of data were recorded continuously during four discrete periods: (1) baseline – prior to placement of the SV in-line with mechanical ventilation; (2) quiet breathing with SV in-line; (3) talking with SV in-line; and (4) post removal of the in-line SV. After the baseline period the tracheostomy cuff was deflated with simultaneous tracheal suctioning to clear secretions pooling above the cuff and minimise aspiration. The SV (PMV007, Passy Muir Inc., Irvine, CA, USA) was then inserted in-line with the ventilation circuit following the adapter that accommodated the EtCO_2_/P_aw_ catheter. Ventilator settings were changed while the SV was in situ in the patients supported by pressure support ventilation (PSV). This included switching the system to non-invasive (NIV) mode for PSV (to more easily control expiratory alarms) and reducing the set ventilator-delivered PEEP by 5 cmH_2_O [25]. This change in settings was based, in the absence of any scientific data to define optimal settings, on recommendations by the SV manufacturer. During the second data collection period patients were instructed to continue to breathe normally and avoid talking. Once the third data collection period commenced the patients were instructed to converse as they wished with the researcher, family member, or healthcare team. When verbal communication was limited, the researcher used picture cards and open-ended questions to facilitate verbal output. As there is a suggested difference in breathing patterns between different speech tasks (planned vs non-planned) [26], no set tasks were given to participants, and spontaneous speech was encouraged. At the completion of period 3, the ventilator settings were returned to baseline, the SV was removed, and the tracheostomy cuff re-inflated. Data collection continued in the fourth period as per baseline conditions. Routine tracheal suctioning was performed during data collection as per individual patient needs.

### Data analysis

Data were analysed offline post data collection using commercially available Draeger software (Draeger EIT Data Analysis Tool 6.1). EELI was averaged across the readings and displayed as mean EELI for each of the four data collection periods. A mixed effects regression model was used to investigate the changes in EELI compared to baseline. Planned comparisons between baseline and each subsequent data collection period were conducted using the paired *t* test, for RR, EtCO_2_, HR and SpO_2_. The level of significance was set at *p* <0.05 throughout, with 95 % confidence intervals quoted where appropriate. All statistical analyses were conducted using STATA_TM_ (version 12.0).

## Results

**Fig. 1:**
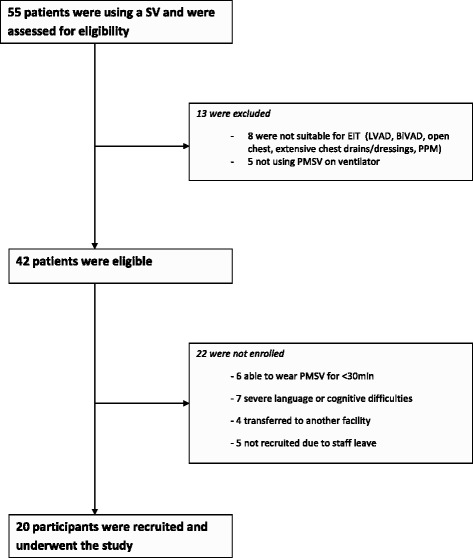
Participant selection chart. *SV* speaking valve, *BiVAD* biventricular assist device, *EIT* electrical impedance tomography, *LVAD* left ventricular assist device, *PMSV* Passy-Muir speaking valve, *PPM* permanent pace maker

During the study period 55 tracheostomised patients used an SV, and all were assessed for inclusion in the study. Of these patients 20 met the inclusion criteria and were enrolled in the study. Figure 1 details the reasons for exclusion or non-participation in the study.

The mean age of the patients in the study was 60.4 ± 14.9 years (50 % male). The mean age for all tracheostomised patients in the ICU throughout the recruitment period was 57.1 ± 17.4 years (64.6 % male). On average, patients used an SV for 2.5 days prior to recruitment to the study. There were 10 patients assessed whilst being ventilated with PSV, and 10 assessed during periods off the ventilator (9 on high-flow, one on low-flow oxygen) for the duration of data collection. All but one of the patients who were assessed off ventilator were still requiring >12 h/day of mechanical ventilation. See Table 1 for the specifics of respiratory requirements. The majority of patients (17) had their tracheostomy tubes inserted percutaneously in the ICU. Primary reasons for ICU admission included cardiac surgery (*n* = 13, 65 %) or respiratory disease (*n* = 5). Nineteen of the patients (95 %) had received a tracheostomy due to prolonged need for mechanical ventilation. Patient number 3 had the tracheostomy initially inserted for surgery in the upper airway, but required prolonged respiratory support following cardiac surgery. See Table 2 for a more detailed description of all patients in the study.

**Table 1:** Participant ventilation needs

Patient number	Vent. needs^a^	Weaned Y/N	PS	PEEP	FiO_2_	Flow
1	HFTP	N	N/A	N/A	40 %	40 L
2	HFTP	Y	N/A	N/A	40 %	40 L
3	LFTP	N	N/A	N/A	30 %	5 L
4	HFTP	N	N/A	N/A	40 %	50 L
5	PSV	N	10	5	40 %	N/A
6	HFTP	N	N/A	N/A	40 %	50 L
7	HFTP	N	N/A	N/A	40 %	40 L
8	PSV	N	15	10	35 %	N/A
9	HFTP	N	N/A	N/A	50 %	50 L
10	PSV	N	13	10	40 %	N/A
11	HFTP	N	N/A	N/A	40 %	40 L
12	PSV	N	10	7.5	40 %	N/A
13	PSV	N	15	5	35 %	N/A
14	HFTP	N	N/A	N/A	30 %	30 L
15	HFTP	N	N/A	N/A	40 %	40 L
16	PSV	N	10	8	40 %	N/A
17	PSV	N	10	5	35 %	N/A
18	PSV	N	12	5	40 %	N/A
19	PSV	N	12	8	45 %	N/A
20	PSV	N	12	7.5	40 %	N/A

^a^respiratory needs at point of recruitment. Considered not weaned if needed mechanical ventilation (*Vent.*) in the preceding 24 h

*FiO*_*2*_ fraction of inspired oxygen, *Flow* O_2_ flow requirements at point of recruitment, *HFTP* high-flow tracheostomy piece (>30 L/min of O_2_), *LFTP* low-flow tracheostomy piece (<30 L/min of O_2_), *PEEP* positive end-expiratory pressure, *PS* pressure support, *PSV* pressure support ventilation

**Table 2:** Demographics and tracheostomy data

Patient number	Age, years	Gender	Primary reason for admission to ICU	Days TT to SV, n	Days to decannulation, n	Insertion method	TT type and size	Days of SV use when recruited, n
1	63	M	acute myocardial infarct; CABG	11	18	perc	long flange Portex 8	2
2	48	F	acute myocardial infarct; tamponade	5	12	perc	cuffed Portex 8	6
3	72	F	Buccal SCC + CABG	5	7	surg	cuffed Portex 7	0
4	71	M	tissue AVR for infective endocarditis	2	4	perc	cuffed Portex 8	1
5	29	M	endarterectomy	2	5	perc	cuffed Portex 8	1
6	77	M	CABG x3 and mechanical AVR	6	23	perc	cuffed Portex 8	1
7	44	F	aortic dissection	6	7	perc	cuffed Portex 8	1
8	33	F	endarterectomy	4	12	perc	cuffed Portex 7	4
9	61	M	H1N1, ARDS	12	23	perc	cuffed Portex 8	8
10	70	M	CABGx2	3	5	perc	cuffed Portex 8	1
11	70	F	cardiac tamponade	4	6	perc	cuffed Portex 7	1
12	43	F	PE	2	5	perc	cuffed Portex 7	2
13	47	F	Influenza A ARDS	4	6	perc	cuffed Portex 8	1
14	70	F	CAP	2	7	perc	cuffed Portex 8	5
15	58	M	CAP	3	N/A	surg	cuffed Portex 8	1
16	62	F	CAP	2	6	perc	cuffed Portex 8	1
17	74	F	extensive GI surgery	10	31	perc	cuffed Portex 7	7
18	78	M	CABG x4	3	5	perc	cuffed Portex 8	2
19	60	M	chest trauma	7	12	surg	long flange Portex 8	2
20	77	M	repeat sternotomy for tissue AVR, CABGx1	4	13	perc	cuffed Portex 8	2

*M* male, *F* female*, SV* speaking valve, *ARDS* acute respiratory distress syndrome, *AVR* aortic valve repair, *CABG* coronary artery bypass graft, *CAP* community acquired pneumonia, *GI* gastrointestinal, *PE* pulmonary embolism, *perc* percutaneous, *SCC* small cell carcinoma, *surg* surgical, *TT* tracheostomy tube

**Fig. 2:**
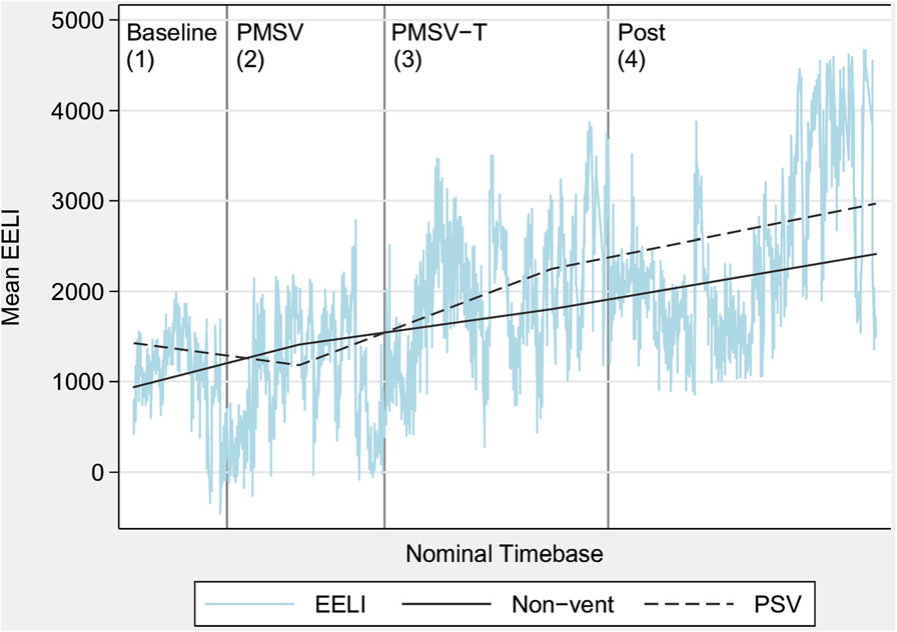
Mean end-expiratory lung impedance (EELI) vs time with average EELI trend for non-vent and pressure support ventilation (PSV). Mean EELI is plotted on the *y-axis* against a nominal time base. A *lowess smoothing line* has been added to clarify the overall trend. *non-vent* patient off mechanical ventilation during recruitment, *SV* speaking valve

A statistically significant increase in EELI was observed between baseline and all subsequent data collection periods. A mean increase by 19.7 % (213 units) occurred from baseline to period 2 (SV + quiet breathing, *p* = 0.034). Further increase from baseline by 83.6 % (905 units) (*p* <0.001) and 120 % (1,299 units) (*p* <0.001) were seen in data collection period 3 and 4, respectively (see Fig. 2 and Table 3).

**Table 3:** Outcome measures across four time periods

	Baseline (1)	SV (2)	SV-talk (3)	Post SV (4)
SpO_2_	96.5 (0.5)	95.5 (0.7)	94.7 (0.7)	96.0 (0.8)
RR	25 (1.6)	22 (1.5)*	20 (1.7)*	25 (1.4)
HR	95 (2.8)	95 (2.4)	96 (2.9)	96 (3.0)
EtCO_2_	29 (1.1)	27 (1.1)*	26 (1.2)*	28 (1.0)
EELI, mean	1082 (57)	1295 (61)*	1987 (60)*	2381 (75)*

All data are presented as mean (standard error of the mean)

*Statistically significant change, *p* <0.05

*EtCO*_*2*_ end-tidal carbon dioxide, *HR* heart rate, *EELI* end-expiratory lung impedance, *RR* respiratory rate, *SpO*_*2*_ peripheral capillary oxygen saturation, *SV* speaking valve

Of note, patients’ ventilatory requirements at the time of recruitment did not have a significant impact on change in EELI, any of the respiratory parameters or HR. The patients who were supported on PSV during data collection had an initial non-significant drop in EELI. However, a similar increase in EELI with patients off the ventilator was noted for the third and fourth period of data collection (see Fig. 2).

EtCO_2_ decreased significantly during SV use (*p* = 0.02 for period 2 and *p* = 0.01 for period 3) and returned to baseline for period 4. RR decreased significantly from baseline while SV was used in-line with the ventilation circuit (*p* = 0.001, *p* <0.001 for periods 2 and 3, respectively), and returned to baseline once the SV was removed. HR and SpO_2_ did not change significantly throughout data collection.

Only limited data on P_aw_ were captured (three participants with full data, seven with partial data). These data all indicated similar drops in P_aw_ coinciding with the reduction of ventilator-delivered PEEP for the duration of the SV use. Ventilator data showed that there was minimal expired tidal volume when the SV was in-line (see Table 4).

**Table 4:** Airway pressure (P_aw_), expired tidal volume (TV) and peak inspiratory pressure (PIP)

	Baseline (1)	SV (2)	Post SV (4)
P_aw_, (*n* = 7)^a^	10.5 cmH_2_O	5.6 cmH_2_O	10.7 cmH_2_O
TV, L (*n* = 10)^b^	0.550	0.024^c^	0.534
PIP (*n* = 10)^b^	19.8	15.1	20

^a^Full data for all three periods from three patients only

^b^Data from all 10 mechanically ventilated patients in the study

^c^Two patients had higher TV of 0.106 L and 0.088 L on average, and two patients had TV of 0.0 L. *SV* speaking valve, P_aw_ airway pressure, TV tidal volume

## Discussion

The findings indicated that use of SVs in this cohort did not result in any significant de-recruitment of the lungs, which was contrary to concerns initially voiced by physicians. Standard bedside respiratory data demonstrated reduced work of breathing with adequate gas exchange. The increase in EELI may indicate increased EELV. Further analysis is necessary to more fully determine ventilation distribution, as an increase in EELV could be due to further recruitment or over-inflation of already aerated parts of the lung.

The increase in EELI with the SV in the ventilator circuit is likely to occur through the restoration of the patient’s ability to breathe through the larynx and upper airway, as opposed to the continuously patent tracheostomy tube. Upper airway resistance is increased due to the resistance created by exhalation against and around the effectively closed tracheostomy tube (through the actions of the SV) and its deflated cuff, ensuring more residual air in the lungs at the end of expiration. Further analysis is required to confirm that lung hyperinflation did not occur as it could be argued that an increase in EELI may correlate to tidal hyperinflation. We used SpO2 and EtCO_2_ as simple measures to exclude pathological degrees of hyperinflation, but this cannot exclude it fully. Of note, all patients had been using an SV before the study with no gross signs of hyperinflation on routine chest radiographs.

The subsequent increase in EELI when patients talked is explicable through the additional, but variable, upper airway resistance caused by the glottis [27] with vocal folds closing and opening during attempts at phonation. The SV appeared to act as a recruitment manoeuvre. An increase in EELI was observed during SV use and its effect remained after removal of the SV from the patient’s ventilation circuit. EELI remained stable for 8–9 minutes once the SV was removed from the ventilator circuit and the tracheostomy cuff re-inflated before a further increase occurred.

There are several potential explanations for the drop in EtCO_2_ during the SV use. One reason may be a drop in EtCO_2_ due to using one’s voice, as observed in a study of healthy subjects [28]. Another potential reason is dead-space washout in the upper airway that has been found in other studies [29, 30] to coincide with an increase in tidal volumes. With our current data, we cannot categorically state, however, that tidal volumes increased for patients in this study. A third potential aetiological cause may be that the exhaled air just past the tracheostomy cannula from where EtCO_2_ was measured was being diluted with fresh inspiratory flow in all patients on high-flow oxygen, and some on PSV while the cuff was deflated. Transcutaneous carbon dioxide (TcCO_2_) and arterial pressure of carbon dioxide (PaCO_2_) may need to be measured in similar studies in the future.

Only limited data on P_aw_ were captured, due to rapid and repeated obstruction of the fine-bore catheter with secretions due to presence of no flow through the catheter during the numerous 2-minute measurements. A similar reduction in P_aw_ coinciding with the turning down of the set ventilator-delivered PEEP for the duration of the SV use was noted. However, due to lack of data, it is difficult to draw any conclusions. Further studies are needed to further look at P_aw_ and ventilator-delivered PEEP with and without an SV in circuit.

It was surprising to observe that the ventilator demonstrated substantial exhaled tidal volume whilst the SV was in situ. This may indicate the presence of a leak in the SV or some form of back-pressure. This means that the ventilator may actually still be delivering PEEP when a one-way valve is in place, and will be the subject of further studies.

Communication is a key issue for ventilated patients, who find the inability to speak distressing [31–33]. Difficulties with communication in the tracheostomised patient population have been associated with social withdrawal, leading to depression, lack of motivation to participate in care [31, 34–36], poor sleep, and increased anxiety and stress levels [37], which has both short-term and long-term impacts on patient outcomes in ICU and post ICU stay. By demonstrating the potential physiological benefits on top of the already known and more obvious psychological benefits, SVs present an excellent way to improve patient care in the ICU.

Increased use of SV brings with it multiple questions, such as, for how long should the SVs be used at any one time? Does this lead to fatigue? Should the SVs be used with patients during mobilisation? Future studies are needed to look at the efficacy of SVs in the weaning and rehabilitation process of mechanically ventilated tracheostomised ICU patients.

### Limitations of the study

This study was conducted on a specific cohort of ICU patients, mostly cardiothoracic, and extrapolation of these data to patients with different pathological conditions may not be wise. This is even more relevant in patients with spinal and brain injuries in whom central control of breathing might be affected.

No patients in this study were ventilated using volume-controlled modes, hence there is a need to determine whether restored physiological PEEP through the SV helps compensate for the leak in the ventilatory circuit similarly in volume-controlled ventilation.

Airway pressures were only measured for the second half of the study with limited data obtained as described above. Hence the reported P_aw_ data may be a poor representation of the actual P_aw_ across the time points in the study, and was therefore not reported in detail. Different methods to obtain these important data are recommended for future similar studies. Minor difficulties also occurred with EtCO_2_ measurements (measured in all patients in the study) through the same catheter. However, due to the presence of airflow in the catheter during EtCO_2_ measurement, this reduced the likelihood of the catheter becoming blocked with secretions, and resulted in almost full data collection across 60 minutes obtained from all patients.

Routine suctioning was performed as per patient needs throughout data collection. It is known that tracheal suctioning causes a degree of de-recruitment [22]. The quantitative effect of suctioning was not specifically analysed as part of this study, nor were these periods excised from data analysis. De-recruitment caused by tracheal suctioning could therefore be a confounding factor and negatively skew our data on the effect of SVs.

The duration of the study was only a total of one hour with the SV in situ for 30 minutes. Clinically the same patients would be using the SV for several hours at a time. Due to the inability to compare the change in EELI between sessions and the patients needing to remain in the same position, the EIT belt stayed in situ for the duration of the study with the patients sitting up. Therefore it was not feasible to monitor the patients for longer.

## Conclusions

When SVs were used in this cohort of cardio-respiratory patients, we observed no evidence of lung de-recruitment whilst weaning from mechanical ventilation. Deflation of the tracheostomy cuff with restoration of the airflow via the upper airway with a one-way valve facilitated an increase in EELI both during and after a period of SV use in our cohort of patients, which may indicate recruitment of the lungs. Use of the SV resulted in reduced RR and a reduced end-tidal CO_2_.

## Key messages

- Speaking valve use facilitated an increase in end-expiratory lung impedance in tracheostomised cardiothoracic ICU patients weaning off mechanical ventilation
- Increased end-expiratory lung impedance was maintained and further increased for at least 15 minutes post removal of the speaking valve from the ventilation circuit
- Speaking valve use resulted in a reduced respiratory rate and reduced end-tidal CO_2_ when used in tracheostomised cardiothoracic ICU patients weaning off mechanical ventilation

## Abbreviations

EELI: end-expiratory lung impedance
EELV: end-expiratory lung volume
EIT: electrical impedance tomography
EtCO_2_: end-tidal carbon dioxide
FiO_2_: fraction of inspired oxygen
HFTP: high-flow tracheostomy piece (as defined by >30 L of continuous O_2_ via tracheostomy tube)
HR: heart rate
ICU: intensive care unit
LFTP: low-flow tracheostomy piece as defined by <30 L of continuous O_2_ via tracheostomy tube)
P_aw_: airway pressure
PEEP: positive end-expiratory pressure
PIP: peak inspiratory pressure
PSV: pressure support ventilation
RR: respiratory rate
SpO_2_: peripheral capillary oxygen saturation
SV: speaking valve
TV: tidal volume
